# Conservative Management of Uterine Adenomyosis: Medical vs. Surgical Approach

**DOI:** 10.3390/jcm10214878

**Published:** 2021-10-22

**Authors:** Christina Anna Stratopoulou, Jacques Donnez, Marie-Madeleine Dolmans

**Affiliations:** 1 Pôle de Recherche en Gynécologie, Institut de Recherche Expérimentale et Clinique, Université Catholique de Louvain, 1200 Brussels, Belgium; christina.stratopoulou@uclouvain.be; 2 Société de Recherche pour l’Infertilité, 1150 Brussels, Belgium; jacques.donnez@gmail.com; 3 Université Catholique de Louvain, 1200 Brussels, Belgium; 4 Gynecology Department, Cliniques Universitaires Saint-Luc, 1200 Brussels, Belgium

**Keywords:** uterine adenomyosis, infertility, heavy menstrual bleeding, dysmenorrhea, medical treatment, GnRH antagonist, conservative surgery

## Abstract

Uterine adenomyosis is a commonly encountered estrogen-dependent disease in reproductive-age women, causing heavy menstrual bleeding, intense pelvic pain, and infertility. Although adenomyosis was previously considered a disease of multiparous women, it is becoming increasingly evident that it also affects younger nulliparous women and may compromise their fertility potential. It is clear that hysterectomy, the standard approach to definitively manage the disease, is not an option for patients wishing to preserve their fertility, so there is an urgent need to develop novel conservative strategies. We searched the current literature for available methods for conservative management of adenomyosis, including both pharmacological and surgical approaches. There is no existing drug that can cure adenomyosis at present, but some off-label treatment options may be used to tackle disease symptoms and improve fertility outcomes. Adenomyosis in patients wishing to conceive can be ‘treated’ by conservative surgery, though these procedures require highly experienced surgeons and pose a considerable risk of uterine rupture during subsequent pregnancies. While currently available options for conservative management of adenomyosis do have some capacity for alleviating symptoms and enhancing patient fertility perspectives, more effective new options are needed, with gonadotropin-releasing hormone antagonists showing encouraging results in preliminary studies.

## 1. Introduction

Uterine adenomyosis is a chronic estrogen-dependent condition affecting approximately 20% of gynecology patients [1]. Its most common symptoms include heavy menstrual bleeding, intense pelvic pain, and infertility. Despite the high prevalence and severe symptoms of the disease, its pathogenesis is not yet fully understood [1,2]. A lack of critical information on the origin of the disease and the absence of a universal classification system create a kind of therapeutic anarchy, with approximately 82% of adenomyosis patients eventually resorting to hysterectomy, a somewhat extreme way to deal with the disease [3,4,5].

We searched the PubMed database (https://pubmed.ncbi.nlm.nih.gov/, accessed during 2020 and 2021 until 11 July 2021) for English-language articles focusing on adenomyosis, its main symptoms (menorrhagia/heavy menstrual bleeding, dysmenorrhea, pelvic pain, infertility), and treatment options. Key words and phrases included ‘medical’ or ‘pharmaceutical’ or ‘surgical treatment,’ as well as specific names of drug categories and procedures. Reference lists of studies of interest were also screened for potentially matching articles.

Our search revealed that there is as yet no drug specifically registered to treat adenomyosis, but various off-label treatments have emerged over the years, some of them with acceptable efficacy [6]. On the other hand, conservative surgical approaches have been developed, ranging from partial reduction surgery to complete adenomyosis excision, but these methods remain a source of controversy, primarily due to the risk of uterine rupture during subsequent pregnancies. The choice of suitable therapy depends on individual patient age, severity of symptoms, and reproductive status, and can vary from full-scale hysterectomy to medical management of clinical symptoms and infertility [6]. A number of small studies have been conducted, but are still insufficient to determine universal management guidelines. The specific medical and surgical approaches we identified and their associated outcomes based on published reports are presented below.

## 2. Medical Treatment of Adenomyosis

Currently available medical therapies for adenomyosis aim to alleviate local hyperestrogenism and tackle the most severe symptoms of adenomyosis, namely heavy menstrual bleeding, dysmenorrhea, and non-menstrual pelvic pain. Little is known about the exact causes underlying adenomyosis-related infertility, but pharmacological management protocols have been developed with favorable results, as discussed further in this review. Here, we explore the most popular medical treatments used to manage the disease. A summary of the advantages and drawbacks of each of these medications can be found in Table 1.

### 2.1. Non-Steroidal Anti-Inflammatory Drugs

Non-steroidal anti-inflammatory drugs (NSAIDs) are the most frequently prescribed class of drugs to treat dysmenorrhea, especially primary dysmenorrhea, showing acceptable efficiency but being prone to adverse side effects when used extensively [7,8]. NSAIDs block cyclooxygenase, suppress prostaglandin, and alleviate uterine hypercontractility, so they relieve pain without treating potential underlying causes, as in case of adenomyosis and endometriosis [8]. Regarding heavy menstrual bleeding, one systematic review concluded that NSAIDs are more effective at treating this particular symptom compared to placebo, but not as effective as other drug categories like progestins [9]. Despite their questionable effectiveness and inability to treat the underlying pathogenic mechanisms of adenomyosis, the non-hormonal nature of NSAIDs might render them an attractive option for women with mild symptoms seeking to conceive.

### 2.2. Combined Oral Contraceptives

Combined oral contraceptives (COCs) act by inhibiting follicle-stimulating hormone (FSH) and luteinizing hormone (LH), subsequently blocking follicle development and endometrial proliferation, which is why they were proposed to treat adenomyosis-related symptoms [10]. To the best of our knowledge, two studies have investigated their use in the clinical management of adenomyosis, comparing them with other available options [11,12]. According to Shaaban et al., a 6-month treatment with COCs did alleviate pain in adenomyosis patients, but both pain and uterine volume showed more pronounced decrease in the levonorgestrel-releasing intrauterine device (LNG-IUD)-treated group [11]. Consistent with these findings, Hassanin et al. found COCs to be effective at diminishing pain, while dienogest was superior at reducing bleeding and uterine volume, but at the cost of more side effects [12]. It should be noted, however, that extensive use of COCs has been linked to increased risk of thromboembolic events [13].

### 2.3. Progestins

There is ample evidence on the use of oral and systemic progestins for treatment of adenomyosis and endometriosis. Progestins exert a broad range of contraceptive effects, including on the endometrium, which they render non-receptive to embryos [14]. They have become popular in adenomyosis management thanks to their ability to suppress secretion of FSH and LH, ultimately inhibiting ovarian steroid formation and allaying adenomyosis-related hyperestrogenism [15].

Numerous clinical trials have focused on use of dienogest, an oral progestin, most of them with encouraging results. However, a pilot study in 15 patients receiving dienogest for 24 weeks failed to demonstrate significant reduction in either uterine or lesion size or the width of the junctional zone, though pain relief levels were acceptable [16]. Moreover, anemia due to metrorrhagia was observed in one-third of patients receiving the treatment. Indeed, a number of clinical trials on long-term use of dienogest in women with dysmenorrhea have reported metrorrhagia as the most common side effect, occurring in almost all subjects at varying severity [17,18]. A shorter course of dienogest therapy (16 weeks) was still associated with abnormal menstrual bleeding, but not severe anemia in most patients [19]. According to a small retrospective study, patients with intrinsic adenomyosis (based on the classification of Kishi et al. [20]) may be more prone to developing serious menorrhagia upon dienogest administration than other disease subtypes [21]. Nevertheless, current data are insufficient to determine whether the subtype of adenomyosis can predict the severity of side effects. 

Similar results have been obtained with subcutaneous progestin (etonogestrel), particularly a clear reduction in pain symptoms, but vaginal bleeding caused several patients to have their implants removed [22,23,24]. Decreased uterine volume, an important parameter linked to menorrhagia, may or may not be achieved in adenomyosis patients treated with etonogestrel, depending on the study [23,24].

The LNG-IUD, an intrauterine version of progestin, has also been proposed to manage symptomatic adenomyosis, yielding satisfactory outcomes, but with some debate around appropriate treatment durations [11,25,26,27,28,29,30]. One clinical trial involving 47 patients reported pain relief from the sixth week after insertion, and a significant decrease in mean uterine volume by month 12 [28]. In the same study, however, it was noted that efficacy declined over time, with pain and uterine volume increasing two years after insertion. On the contrary, another trial demonstrated more long-term effects of the LNG-IUD against adenomyosis symptoms, as both dysmenorrhea and uterine volume remained significantly reduced after 3 years of follow-up of 94 enrolled patients [29]. A more recent retrospective study suggested that the beneficial impact of LNG-IUDs on heavy bleeding and dysmenorrhea, but not uterine volume reduction, may last up to six years post-insertion [30].

The major disadvantage of progestin use is that approximately one-third of patients do not respond to these drugs, most likely due to progesterone resistance [15,31,32]. As in endometriosis, progesterone receptors in adenomyotic uteri are either diminished or inactive, thus inhibiting the action of both local and synthetic progesterone forms [1,15,31,32]. As also described in our previous review article, other important limitations of progestins include doubtful efficacy in reducing uterine volume and possible links to venous or arterial embolism events [15]. Taken together, progestins constitute a common and acceptable option for managing adenomyosis symptoms, but the high percentage of non-responders highlights the need to develop new options.

### 2.4. Ulipristal Acetate

**Figure 1:**
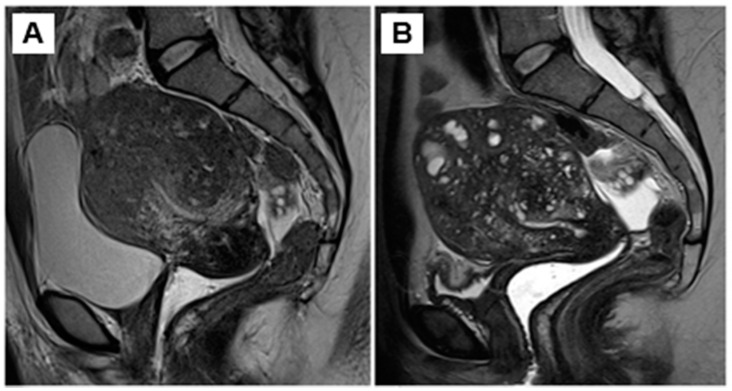
(**A**) MRI showing an enlarged uterus with diffuse and disseminated adenomyosis. (**B**) Worsening of MRI features after therapy with UPA. Numerous spots typical of adenomyosis are visible: asymmetric heterogeneous myometrium with multiple myometrial cysts, related to dilated islets of ectopic endometrium.

Ulipristal acetate (UPA) is a selective progesterone receptor modulator currently registered for specific clinical indications: emergency contraception and long-term management of symptomatic uterine fibroids or prior to myoma surgery [33,34]. It is capable of delaying ovulation and endometrial maturation by lowering serum estradiol levels [35]. Based on these properties, UPA has been tested in managing adenomyosis symptoms, but only limited clinical evidence supports its use. Two recent studies reported a diminution in blood loss after a 12-week treatment with UPA in 30 patients with adenomyosis and 41 patients with concurrent adenomyosis and leiomyoma [36,37]. Conversely, three studies have recorded worsening of symptoms and imaging features of adenomyosis, suggesting that it may be time to abandon this option [38,39,40]. Donnez and Donnez recently reported aggravation of symptoms and magnetic resonance imaging (MRI) features of adenomyosis after a 3-month course of UPA (Figure 1) [38]. Similarly, Conway et al. demonstrated exacerbation of ultrasound characteristics of adenomyosis in women inappropriately treated with UPA for leiomyomas, observing enlarged intramyometrial cysts and enhanced vascularization [39]. Most patients also experienced increased pain after treatment, including pelvic pain, dyspareunia, and bowel symptoms, leading the authors to emphasize the importance of accurate diagnosis of adenomyosis, and effectively dismissing UPA as a credible treatment for the disease [39]. Finally, Calderon et al. observed progression of adenomyosis by MRI as early as three months after UPA treatment initiation in patients with concurrent leiomyomas [40].

### 2.5. Gonadotropin-Releasing Hormone (GnRH) Agonists

GnRH agonist competitively binds to the natural GnRH receptors, but has a longer life span than the endogenous peptide [41]. Its use in the context of adenomyosis relies on its anti-proliferative effects in the myometrium, following the suppression of gonadotropin secretion and the subsequent drastic decline in estradiol [6]. The efficacy of GnRH agonist at reducing uterine volume, inducing amenorrhea, and relieving pain was demonstrated by case reports as far back as three decades ago [42,43]. It was subsequently shown that a 6-month course of GnRH agonist was able to significantly reduce both uterine volume and JZ thickness in 18 adenomyosis patients, some of whom had concurrent endometriosis [44]. Its effect on chronic pelvic pain symptoms also appears considerable, curbing pain medication use and curtailing lost work productivity [45].

Despite these promising results, GnRH agonists are associated with severe hypoestrogenic side effects, especially a reduction in bone mineral density, so they cannot be used long-term, while symptoms tend to reappear upon treatment cessation [13,46]. Add-back therapy is helpful in decreasing this effect, but experts in the field have argued that long-term treatment of adenomyosis with GnRH agonist should be limited to cases not responding to other medications or where surgical intervention is not a safe option [6,47]. On the other hand, pre-treatment with GnRH agonist in infertile patients seeking undergoing assisted reproduction may indeed be a viable option, as discussed further in this review [48].

### 2.6. Oral GnRH Antagonists

**Figure 2:**
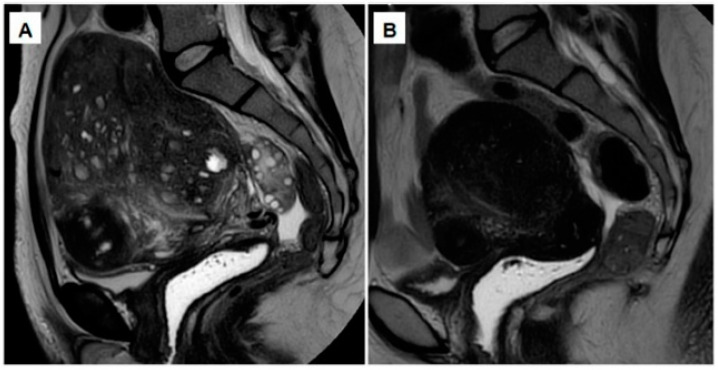
(**A**) MRI showing an enlarged uterus, consistent with severe full-thickness adenomyosis. (**B**) After a 12-week course of GnRH antagonist (200 mg linzagolix daily), a reduction can be observed in both uterine size and adenomyotic foci.

While not yet registered for clinical use against symptomatic adenomyosis, oral GnRH antagonists constitute an attractive option, increasingly gaining the interest of the medical community and yielding promising results in preliminary studies [49,50]. These molecules antagonize GnRH binding to its receptors, leading to dose-dependent suppression of FSH and LH and a subsequent decline in ovarian steroids [15,41]. The major advantages of these drugs over GnRH agonists are: (i) their water-soluble nature, allowing easy and tolerable oral administration; (ii) rapid action without the initial 1–2 weeks of the flare-up effect; (iii) dose-dependent and easily reversible modulation of the hypothalamic-pituitary-gonadal axis; and (iv) incomplete suppression of FSH and LH when administered at low doses, hence avoiding related side effects [15]. Early data indeed demonstrated their effectiveness in lesion regression, reduction of uterine volume, and alleviation of clinical symptoms in a patient with severe adenomyosis [38] (Figure 2). The results of a more recently published study [51] further provide evidence that linzagolix administered at a high dose (200 mg daily) for 12 weeks, followed by a lower maintenance dose (100 mg daily) for 12 weeks, substantially reduced uterine volume and adenomyotic lesions, as well as adenomyosis-related pain. Future studies will determine the advantages of continuing the intake of linzagolix 100 mg daily after week 24 to avoid regrowth of the uterus [51]. Moreover, Borini and Coticchio discussed the potential superiority of GnRH antagonist over GnRH agonist in patients wishing to preserve their fertility, primarily because it does not radically suppress circulating LH, and its impact on the hypothalamic-pituitary-gonadal axis can be easily reversed [52]. This is a major advantage in the context of infertility management and, as described in a recent review paper, would entail oocyte retrieval and vitrification followed by a 12-week course of treatment to reduce lesion size and uterine volume prior to frozen-thawed embryo transfer [53]. Ongoing and future clinical trials are expected to confirm the efficacy and safety of GnRH antagonists against symptomatic adenomyosis and its potential benefits for infertile patients.

Despite limited data in the context of adenomyosis, oral GnRH antagonist-based elagolix and linzagolix have proved efficient in treating endometriosis-related pain and improving patient quality of life [54,55,56,57]. Moreover, although one trial showed loss of bone mineral density at higher doses, add-back therapy could be avoided with the 75 mg dose, while still maintaining the treatment’s beneficial effects [55]. Consistent with these findings, an ongoing clinical trial has reported that relugolix, another drug in the same category, can also successfully reduce endometriosis-associated pain when administered for up to 24 weeks at a dose of 40 mg [58]. Indeed, we have already stressed that adenomyosis and endometriosis, particularly deep endometriosis, frequently coexist and may as well share the same pathogenic origin, suggesting the potential value of extrapolating treatment results from one condition to the other [59,60].

### 2.7. Experimental Approaches and Preclinical Models

#### 2.7.1. Bromocriptine

A variety of medical treatments for adenomyosis have been proposed primarily based on findings from preclinical models, but have served little or no clinical purpose so far. One such treatment is bromocriptine, a dopamine agonist frequently prescribed to manage hyperprolactinemia. Its use is based on the observation that prolactin is aberrantly expressed and may induce an adenomyosis-like phenotype in mouse models [61,62]. In studies by Andersson et al., a 6-month course of vaginal bromocriptine was found to significantly reduce adenomyosis-associated pain and JZ thickness in a small number of patients [63,64]. Uterine bleeding also declined post-treatment but remained high [63]. Indeed, high serum prolactin has been linked to endometriosis-related infertility, but a strong causal link between this protein and adenomyosis or endometriosis has not yet been identified [65]. The association of adenomyosis and aberrant prolactin signaling is almost exclusively corroborated by findings in a mouse model, so its mechanisms of action in human adenomyosis development and symptoms are unclear [65].

#### 2.7.2. Aromatase Inhibitors

Aromatase inhibitors have also been theoretically proposed, but have not yet found widespread clinical application in the context of adenomyosis. In one case report, concomitant administration of an oral aromatase inhibitor (anastrozole) and a GnRH agonist (goserelin acetate) was successful in reducing uterine volume and inhibiting bleeding in a patient with endometriosis and severe adenomyosis, who was resistant to progestin [66]. To the best of our knowledge, only one prospective study involving 32 patients has analyzed the effectiveness of letrozole compared to GnRH agonist in treating adenomyosis [67]. A significant reduction in uterine volume was observed post-treatment in both study groups, with GnRH agonist emerging as more effective in the fourth and eighth weeks of treatment. It was also superior in relieving menorrhagia, dysmenorrhea, dyspareunia, and non-menstrual pelvic pain. All in all, data supporting use of aromatase inhibitors against adenomyosis symptoms remain limited and inconclusive, with endometrial and ectopic lesion production of aromatase still a matter of debate [68,69,70].

#### 2.7.3. Anti-Platelet Therapy

One mouse model-based study suggested the potential utility of ozagrel, an anti-platelet drug, for treatment of adenomyosis, after observing enhanced platelet aggregation in mouse and human lesions [71,72]. More specifically, anti-platelet therapy appeared to lessen uterine contractility, hyperalgesia, and immune cell infiltration in an adenomyosis mouse model [71]. It should, however, be pointed out that the potential impact of such treatment on heavy menstrual bleeding cannot be investigated in non-menstruating mice. No data are yet available on the possible effect of ozagrel or other anti-platelet agents on human adenomyosis, while the actual role of platelet aggregation in its pathogenesis remains contentious [60,73].

#### 2.7.4. MicroRNAs

Developing a novel drug targeting adenomyosis-related signaling pathways without affecting nearby tissues certainly sounds appealing. Indeed, microRNA therapeutics are increasingly attracting the attention of the scientific community thanks to their unique ability to specifically target and simultaneously silence multiple genes, including genes crucial to human disease development. In theory, restoring expression of selected dysregulated microRNAs would result in transcriptional regulation of their downstream pathways and, hence, disease treatment. It has been demonstrated that several microRNAs are dysregulated in endometrium from adenomyosis patients, raising hopes of developing a treatment by normalizing expression of these molecules [74]. According to subsequent studies, let-7a, miR-145-5p, miR-17, and miR-2861 have all been found to be differentially expressed in adenomyosis and may constitute targets for development of novel therapies against the disease [75,76,77,78]. Nevertheless, there is still a huge gap to bridge between in vitro studies and development of a drug for clinical application, with the first ever small RNA-based therapeutic (patisiran) only obtaining FDA approval as recently as 2018.

### 2.8. Medical Management of Infertility in Adenomyosis Patients

Adenomyosis has been repeatedly associated with both poor in vitro fertilization (IVF) outcomes and pregnancy complications [79,80]. The underlying mechanisms remain unclear, but a variety of factors have been suspected of contributing to adenomyosis-related infertility. Uterine hypercontractility, a hallmark of adenomyosis, may inhibit physiological sperm transport through the female reproductive tract, thus inhibiting oocyte fertilization [81]. Impaired stromal decidualization may constitute another reason, given the frequently encountered resistance to progesterone in adenomyosis patients, a hormone playing a pivotal role in the transition of endometrium to its decidualized state [1,15,82,83]. On the other hand, the chronic inflammatory endometrial environment may be the underlying cause of adenomyosis-related infertility, as it is hostile to embryos [84]. Treating adenomyosis-related hyperestrogenism, hypercontractility, and local inflammation, therefore, appears to be crucial to enhancing patient fertility.

A number of studies have suggested the possible utility of GnRH agonist pre-treatment in infertile adenomyosis patients undergoing IVF. In fact, according to a study of 45 subjects, triptorelin with add-back therapy may be beneficial to infertile patients irrespective of IVF, by reducing uterine size and enhancing elasticity, hence facilitating spontaneous pregnancy [85]. Several case reports have further demonstrated effectiveness in patients with severe adenomyosis suffering from secondary infertility [86,87,88]. One of these women later presented with severe pregnancy complications due to insufficiently decidualized endometrium, but it was not clear whether this event was related to the effect of GnRH agonist [88]. Niu et al. reported a significant improvement in rates of clinical pregnancy, successful implantation, and ongoing pregnancy in 339 infertile patients with adenomyosis undergoing treatment with GnRH agonist prior to frozen embryo transfer [48]. A more recent study, however, failed to identify a significant difference in clinical pregnancy rates between 241 treated and untreated infertile adenomyosis patients in this category [89]. Two studies concluded that conservative surgery combined with GnRH agonist, rather than GnRH agonist alone, is able to improve symptoms and boost reproductive potential in adenomyosis patients [90,91]. Finally, an ultra-long GnRH protocol before ovarian stimulation appears to yield better pregnancy outcomes than the corresponding long protocol [92,93]. More specifically, one study reported significantly higher pregnancy and live birth rates in patients with diffuse adenomyosis receiving a 3.75 mg dose of diphereline subcutaneously on a monthly basis for 2–4 months prior to stimulation compared to corresponding patients receiving a single 0.93–1.87 mg dose on the 18th–20th day [92]. Consistent with these findings, a second study reported a significant increase in rates of both clinical pregnancy and live births in adenomyosis patients pre-treated with triptorelin (3.75 mg) intramuscularly every 28 days for at least 3 months, compared with 0.1 mg/day dose for 10 days [93].

## 3. Surgical Management of Adenomyosis

The first conservative surgical therapy for adenomyosis in young women, namely adenomyomectomy, dates all the way back to 1952 [94]. In the 1970s, cytoreductive surgery in the form of partial excision of adenomyotic tissue became more popular [95]. Common approaches to conservative partial or complete adenomyosis excision are summarized below.

### 3.1. Partial Reduction Surgery

#### Wedge Resection

Wedge resection involves identifying the seromuscular layer where the adenomyoma is located, before removing parts of the affected serosa and uterine adenomyosis. In 1991, a modification to partial adenomyosis excision was reported, which entailed cutting the adenomyomatous tissue into thin slices using a microsurgical technique [95]. A transverse H incision was then made on the uterine fundus. After removal of the adenomyotic tissue, the myometrial edges were sutured to ensure hemostasis in one or two layers. Serosal flaps resulting from the vertical and transverse incisions were closed with interrupted subserosal sutures. In a study by Fujishita et al., 41 patients underwent the H incision technique and 31 attempted to conceive [96]. Twelve women became pregnant of whom 5 (16.1%) miscarried, leaving 7 (22.5%) reported live births.

### 3.2. Complete Adenomyosis Excision

#### 3.2.1. The Triple-Flap Method

**Figure 3:**
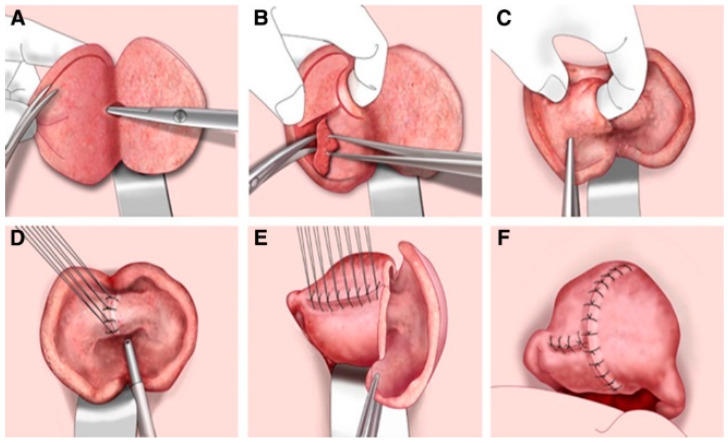
Schematic representation of the triple-flap method applied to the anterior and posterior uterine wall. The approach involves complete extraction of the uterine adenomyosis (**A**–**C**), followed by reconstruction of the uterine cavity (**D**–**F**). An endometrial uterine muscle flap is created by metroplasty and uterine muscle located on the serosal side is used to fill the large uterine wall muscle defect (**E**,**F**) (reprinted with permission from [95]).

With a view to future pregnancy, the triple-flap method has been proposed as an innovative and safer technique for removal of adenomyosis and reconstruction of the uterine wall [95,97]. The approach involves complete extraction of the uterine adenomyosis, followed by reconstruction of the uterine cavity to allow it to sustain subsequent pregnancy, inside which an endometrial uterine muscle flap is created by metroplasty (Figure 3). Uterine muscle located on the serosal side is used to fill the large uterine wall muscle defect. This technique, therefore, repairs the uterine wall defect using normal uterine muscle and, according to Osada, is effective for both diffuse and nodular adenomyosis, potentially helping to prevent uterine rupture [95]. The triple-flap method is extremely difficult to perform by laparoscopic surgery, so open surgery is preferable. Indeed, removal of adenomyotic tissue is ideally carried out under hand palpation, with careful suture of the myometrial layers. In one study, blood flow in the operated area returned to normal within 6 months in 81.4% of women (*n* = 113) undergoing the triple-flap technique. Of 62 women who wished to conceive, 32 (51%) gave birth to a healthy baby by elective cesarean section [98].

#### 3.2.2. Asymmetric Dissection

The asymmetric dissection method is an alternative approach that involves incision of the uterus longitudinally and dissection of the myometrium diagonally, before making a transverse incision to open the uterine cavity [95]. While inserting the index finger into the uterine cavity, the adenomyotic lesion is excised to >5 mm from the inner myometrium, and then excised to >5 mm from the serosal myometrium on the left uterine side. The uterine cavity is subsequently sutured and closed, followed by uterine reconstruction, with the left side covering the right. Uterine rupture was observed in 5 cases out of a series of 1349 patients undergoing this technique [99,100].

### 3.3. Important Remarks on the Surgical Approach

It should be noted that the boundary between adenomyotic tissue and normal myometrium is typically felt by palpation with fingers, so adenomyotic tissue dissection/resection often requires open surgery with all the risks it entails. However, while these experimental techniques are currently performed via an open approach, it is not inconceivable that these same techniques could be adopted via laparoscopy, especially with the benefits of a robotic surgical platform. On the other hand, due to the absence of a cleavage plane, adenomyotic tissue is sometimes overlooked and repair of the uterine wall becomes difficult. Osada pointed out that forced reconstruction of the uterine wall would strain the tissue abnormally and likely result in ruptured sutures and anastomotic leakage [95]. In the view of many gynecologists, diffuse lesions must be excised by laparotomy, which is also needed for the suture of myometrial tissue, and is far more challenging after adenomyotic resection than after myomectomy. Laparoscopy should be restricted to well delimited and focal adenomyomas.

Most importantly, we require clarification on the elevated risk of uterine rupture during pregnancy after adenomyosis surgery, which is provided in Osada’s review [95]. Uterine rupture is a serious obstetrical complication, sometimes even endangering the life of the patient, and its incidence is much higher after adenomyosis excision than after myomectomy. The surgeon should take care to avoid discoloration and heat denaturation of the incision edges, which would make the suture area more fragile and probably increase the risk of rupture. Giving patients and gyne-obstetricians accurate information and obtaining informed preoperative consent are absolutely vital.

## 4. Non-Surgical Alternatives

Uterine artery embolization (UAE) has been proposed as a less invasive alternative to hysterectomy to treat adenomyosis. During this angiographic procedure, an embolic agent is injected into the uterine arteries to induce ischaemic infarction of adenomyotic foci [101,102]. In an extensive review by de Bruyn et al. [101], it was showed that UAE improves clinical symptoms in 83% of patients, but great JZ thickness is associated with decreased effectiveness. The authors also concluded that there is a lack of strong clinical evidence regarding UAE and randomized controlled trials are essential to determine if this procedure is a valuable alternative to hysterectomy. Indeed, as clarified by the same authors, limitations are numerous and include the retrospective evaluation of data, small sample size, and vague description of methodology [101]. Moreover, no solid conclusion concerning uterine volume can be drawn, and fertility after UAE was not evaluated.

In a more recent study [102], women having undergone UAE for adenomyosis were contacted via SMS, email, or phone calls and invited to participate in the follow-up study on a voluntary basis. Improvement of symptoms was indeed observed in 80% of patients responding to the survey, but it should be stressed that recall bias may limit the accuracy of these findings. Unfortunately, data on uterine volume after UAE are missing from this study as well. Future randomized clinical trials should evaluate if UAE should be offered to women in whom conservative management for adenomyosis has failed as a less invasive alternative to hysterectomy.

## 5. Conclusions

Uterine adenomyosis remains an enigmatic disease and there are currently no widely accepted guidelines on its conservative management. The surgical approach is a source of concern, because of the risk of complications during subsequent pregnancy, pushing us to explore new options. Novel treatment with GnRH antagonist has already yielded encouraging results [14,39,49]. However, appropriate therapy for each patient needs to be carefully determined, taking into account lesion size, severity of symptoms, potential wish to conceive, and presence or absence of infertility. Medical treatment is the option of choice to improve clinical symptoms and partially reverse infertility, especially in milder cases. Conservative surgery may be required when lesions persist, bearing in mind all the associated risks.

## Figures and Tables

**Table 1:** Advantages and drawbacks of the main drug groups used against adenomyosis symptoms.

Medication	Advantages	Drawbacks
NSAIDs	Effective against mild pain	Extensive use linked to side effects
	Non-hormonal composition	Inability to treat underlying causes of pain
	Safe for women wishing to conceive	Questionable effectiveness against HMB
COCs	Relatively effective in relieving pain	Limited efficacy in reducing HMB and uterine volume
	Fewer side effects than other drugs	Risk of thromboembolic events
Progestins	Alleviate local hyperestrogenism	Ineffective in about one-third of patients
	Relieve pain symptoms	Frequently cause metrorrhagia at varying severity
	Possibility of long-term symptom management	Doubtful efficacy in diminishing uterine volume
	Ample evidence in favor of LNG-IUD use	
UPA	Lower serum estradiol levels	Numerous reports of symptom and imaging feature exacerbation
	May reduce HMB	Limited to restricted indications by the European Medicines Agency
GnRH agonists	Alleviate pain	Flare-up effect
	Induce amenorrhea	Severe hypoestrogenic side-effects (i.e., vasomotor syndrome, loss of BMD)
	Reduce uterine volume and JZ thickness	Long-term administration not indicated even with add-back therapy
	Beneficial as pre-treatment in infertile patients	
GnRH antagonists	Easy and tolerable oral administration	Loss of BMD at high doses
	Rapid action skipping initial flare-up	Less efficient when combined with add-back medication
	Effectively reduce HMB and pain symptoms	
	Dose-dependent estradiol suppression with less severe side-effects	

NSAIDs: non-steroidal anti-inflammatory drugs; HMB: heavy menstrual bleeding; COCs: combined oral contraceptives; LNG-IUD: levonorgestrel-releasing intrauterine device; UPA: ulipristal acetate; GnRH: gonadotropin-releasing hormone; JZ: junctional zone; BMD: bone mineral density.

## Data Availability

Not applicable.
